# Health-Promoting Properties of *Lactobacillus helveticus*

**DOI:** 10.3389/fmicb.2012.00392

**Published:** 2012-11-19

**Authors:** Valentina Taverniti, Simone Guglielmetti

**Affiliations:** ^1^Division of Food Microbiology and Bioprocesses, Department of Food Environmental and Nutritional Sciences, Università degli Studi di MilanoMilan, Italy

**Keywords:** Probiotic, *Lactobacillus helveticus*, host-bacteria cross-talk, immunomodulation, ACE inhibitory activity

## Abstract

*Lactobacillus helveticus* is an important industrial thermophilic starter that is predominantly employed in the fermentation of milk for the manufacture of several cheeses. In addition to its technological importance, a growing body of scientific evidence shows that strains belonging to the *L. helveticus* species have health-promoting properties. In this review, we synthesize the results of numerous primary literature papers concerning the ability of *L. helveticus* strains to positively influence human health. Several *in vitro* studies showed that *L. helveticus* possesses many common probiotic properties, such as the ability to survive gastrointestinal transit, adhere to epithelial cells, and antagonize pathogens. *In vivo* studies in murine models showed that *L. helveticus* could prevent gastrointestinal infections, enhance protection against pathogens, modulate host immune responses, and affect the composition of the intestinal microbiota. Interventional studies and clinical trials have also demonstrated a number of health-promoting properties of *L. helveticus*. Finally, several studies suggested that specific enzymatic activities of *L. helveticus* could indirectly benefit the human host by enhancing the bioavailability of nutrients, removing allergens and other undesired molecules from food, and producing bioactive peptides through the digestion of food proteins. In conclusion, this review demonstrates that in light of the scientific literature presented, *L. helveticus* can be included among the bacterial species that are generally considered to be probiotic.

## Introduction

Lactobacilli are strictly fermentative bacteria of the phylum Firmicutes that live in a variety of environmental niches, wherever high levels of nutrients (particularly sugars and organic nitrogen) are present (Hammes et al., 1992). Comparative genome analyses demonstrated that during its evolution, the genus *Lactobacillus* diverged from its ancestors through an extensive loss of genes, which was likely triggered by the transition to nutritionally rich environments (Makarova et al., 2006). Currently, the genus *Lactobacillus* comprises more than 80 different species, most of which can be clustered according to their phylogenetic relationships into seven groups: the *Lactobacillus buchneri* group, the *Lactobacillus casei* group, the *Lactobacillus delbrueckii* group, the *L. plantarum* group, the *L. reuteri* group, the *L. sakei* group, and the *L. salivarius* group (Hammes et al., 1992).

The *L. delbrueckii* group comprises scientifically well-known homofermentative bacteria that are typically isolated from human and animal intestinal tracts (*L. acidophilus*, *L. crispatus*, *L. gasseri*, *L. johnsonii*) or from fermented foods (*L. delbrueckii*, *L. helveticus*). Within the *L. delbrueckii* group, the species *L. acidophilus* and *L. helveticus* are phylogenetically very closely related (the 16S rDNA sequences of these bacteria differ by just 1.6%; Callanan et al., 2008); however, while *L. helveticus* is a specialist dairy culture, *L. acidophilus* is a natural inhabitant of the human gut. Furthermore, *L. acidophilus* strains are commonly used as probiotics, defined by the Food and Agriculture Organization of the United Nations (FAO) as *“live microorganisms which when administered in adequate amounts confer a health benefit on the host*” (Food and Agriculture Organization/World Health Organization, 2002). More than 250 scientific studies have been published concerning the probiotic potential of *L. acidophilus* in less than 3 years (from January 2010 to August 2012, according to a PubMed search), whereas *L. helveticus* has been included in less than 20 studies on probiotics in the same period. Although limited, the literature on the potential beneficial effects of *L. helveticus* includes significant scientific facts that highlight the ability of *L. helveticus* strains to interact with the host and influence health.

Here, we review the scientific literature in which *L. helveticus* cells and cell components, or the enzymatic activities of this bacterium, have been linked to specific effects on host physiology. The purpose of this review is to provide scientific evidence that the species *L. helveticus* includes strains that can be properly considered to be probiotic. To this aim, we examined more than 50 scientific publications on the probiotic properties of *L. helveticus*, including *in vitro* characterizations, *in vivo* animal studies, and interventional/clinical trials. The publications cited in this review demonstrate that members of the species *L. helveticus* can potentially affect human health through direct or indirect mechanisms, such as the inhibition of pathogens, modification of gut microbiota, modulation of the host immune system, generation of bioactive peptides from food molecules, or improvement of food quality.

## Inhibition of Fungal and Bacterial Pathogens: The Role of *L. helveticus* in the Prevention and Treatment of Infections

Infectious diseases represent a perpetual threat to human health. According to the 2004 World Health Report from the World Health Organization (WHO) (2004), infectious diseases are the second-leading cause of death following cardiovascular diseases, responsible for about 26% of the deaths that occurred worldwide in 2002. The acquisition of antimicrobial resistance by pathogens, and the subsequent spreading of resistant microorganisms, is a major health concern. Resistance is mainly caused by the misuse of antibiotics that often are prescribed unnecessarily (Wise et al., 1998). In this context, the use of probiotic microorganisms able to exert an antagonistic activity toward pathogens can represent an alternative intervention to prevent infections and might reduce excessive administration of antibiotics. Several studies have demonstrated that *L. helveticus* is effective in preventing infections. In a study performed by Vinderola et al. (2007a), strain *L. helveticus* R389, which does not adhere to the intestinal epithelium, exerted a protective action against *Salmonella enteritidis* serovar Typhimurium infection in mice. *L. helveticus* was also demonstrated to antagonize *Campylobacter jejuni in vitro*, which is the leading cause of enterocolitis in humans in several countries (Ternhag et al., 2008). *L. helveticus* strain R0052 efficiently reduced the invasion of two different strains of *C. jejuni* in human colon cancer epithelial T84 cells (Wine et al., 2009). Heat-inactivated *L. helveticus* cells were observed to maintain part of the antagonistic activity, suggesting that the capacity of *L. helveticus* to block pathogen invasion is not completely dependent on the presence of metabolically active bacterial cells, and that this capacity could be due to the presence of specific cell components, such as the S-layer protein, on the bacterial surface (Johnson-Henry et al., 2007; Wine et al., 2009).

*Lactobacillus helveticus* was also demonstrated to act synergistically with other bacterial strains to antagonize pathogens. For example, *L. helveticus* strain R0052 was used in combination with *L. rhamnosus* to treat mice infected with *Citrobacter rodentium*, a murine pathogen that mimics the infections caused in humans by enterohemorrhagic *E. coli* (EHEC). Specifically, when C57BL/6 mice were pretreated with a mixture of the two lactobacilli and then infected with *C. rodentium*, the authors observed a reduction in weight loss, colonic epithelial cell hyperplasia, and mucosal barrier dysfunctions, thus resulting in an increased survival of the neonatal mice (Gareau et al., 2010).

The ability of a bacterium and/or its components to specifically inhibit a harmful microorganism might also result in a wider beneficial protective action. Pathogens often compromise host cell defense mechanisms and immune responses to infections caused by other etiological agents (Bhavsar et al., 2007). For instance, EHEC inhibits interferon (IFN)-γ-stimulated tyrosine phosphorylation of signal transducer and activator of transcription-1 (STAT-1) in different cell lines (Ceponis et al., 2003), which is an essential signaling pathway in mounting an innate immune response against several bacterial infections (Shtrichman and Samuel, 2001). In research carried out by Jandu et al. (2009), *L. helveticus* R0052 was demonstrated to counteract *E. coli* O157:H7-induced inhibition of STAT-1 tyrosine phosphorylation on three different epithelial cell lines (407, Caco-2, and HEp cells), whereas *L. rhamnosus* R0011 failed to protect cells against pathogen-induced alterations of innate immunity. Moreover, it was demonstrated that intact viable cells were essential to counteract pathogen activity, since boiled cells, conditioned medium, and culture supernatant did not exert any antagonism.

Single molecules isolated from bacterial cells have been demonstrated to be effective against pathogens. For instance, the S-layer protein purified from *L. helveticus* strain R0052 was able to interfere with the adhesion of *E. coli* O157:H7 onto human epithelial HEp-2 cells (derived from larynx epidermoid carcinoma tissue) and to attenuate the pathogen-induced drop in trans-epithelial resistance in human colon carcinoma-derived T84 cells, therefore preserving barrier integrity and functions (Johnson-Henry et al., 2007). A subsequent study demonstrated that the S-layer protein of *L. helveticus* strain M92 mediates the auto-aggregation of the bacterium and co-aggregation with *Salmonella* Typhimurium FP1, contributing to the competitive exclusion of the pathogen (Beganovic et al., 2011).

Interestingly, *L. helveticus* can display efficient epithelium adhesion and pathogen inhibition in body sites other than the gut. In fact, *L. helveticus* strain MIMLh5, isolated from Grana Padano natural whey starter, adhered efficiently to both the human hypopharyngeal epithelial cell line FaDu and HaCat keratinocytes, and inhibited the adhesion of *Streptococcus pyogenes* (the etiological agent of numerous diseases, including sore throat and acute rheumatic fever) better than the 10 probiotic and dairy lactic acid bacterial strains tested (Guglielmetti et al., 2010b). It was also demonstrated that *L. helveticus* interfered with the adhesion of pathogens onto uro-vaginal surfaces. For instance, *L. helveticus* KS300, a hydrogen peroxide-producing strain isolated from the human vagina, inhibited the growth and reduced the viability of vaginosis-associated bacteria *Gardnerella vaginalis* and *Prevotella bivia*, uropathogenic *E. coli* and diarrheagenic *Salmonella* Typhimurium (Atassi et al., 2006). Moreover, strain KS300 interfered with cell adhesion and the entry of uropathogenic Dr adhesin-positive *E. coli* (Nowicki et al., 1987) and diarrheagenic *S*. Typhimurium in human cervical HeLa and Caco-2/TC7 enterocyte-like cell lines, as well as *G. vaginalis* adhesion onto HeLa cells (Atassi et al., 2006). Furthermore, it was recently shown that *L. helveticus* HY7801 inhibited *Candida albicans*-induced vaginitis in mice more potently than three other lactic acid bacteria (LAB) strains, including *L. delbrueckii* ssp. *Bulgaricus* HY7901, *L. gasseri* HY7022, and *L. fermentum* CS332 (Joo et al., 2012). The use of strain HY7801, both orally and intra-vaginally, decreased myeloperoxidase activity in vaginal tissue, which is normally caused by *C. albicans* proliferation (Joo et al., 2012). It was also demonstrated that *L. helveticus* exerted its protective effect when administered orally. Consequently, it has been suggested that *L. helveticus* may act not only through the production of hydrogen peroxide, lactic acid, or other antimicrobial products, but also by stimulating host immunity at the systemic level. The beneficial effects exerted by *L. helveticus* in the vagina are of particular relevance to recurrent vulvo-vaginal candidiasis, which is at least partially caused by the spread of resistant *C. albicans* strains that can originate from prolonged antimicrobial treatments (Rogers, 2002).

In conclusion, strains of *L. helveticus* have the ability to inhibit different pathogenic microorganisms. Considering its GRAS/QPS status, the consumption of *L. helveticus* cells represents an effective and safe strategy for the prevention of pathogen colonization and proliferation.

## The Impact of *L. helveticus* on Host Microbiota Composition and the Metabolic Profile

Strains belonging to the *L. helveticus* species can exert beneficial effects on the host’s health by positively affecting the microbiota composition of a specific body site. In an *in vivo* study using fecal plate counts in mice, the administration of *L. helveticus* M92 for 8 days increased the levels of total LAB and decreased total enterobacteria and sulfur-reducing clostridia; this effect was particularly evident when strain M92 was used in combination with different prebiotic fibers (especially inulin) in a symbiotic preparation (Frece et al., 2009). The authors stated that the effect exerted by *L. helveticus* on the composition of the gut microbiota might depend on the production of lactic acid and bacteriocins. The same M92 strain was also orally administered for 7 days to Swiss albino mice in combination with *Salmonella* Typhimurium FP1. The administration of *L. helveticus* caused a decrease of both total enterobacterial and *Salmonella* spp. counts by approximately 2 log units, compared to mice infected only with the pathogen (Beganovic et al., 2011). In another study, *L. helveticus* 416 was used in association with *Enterococcus faecium* CRL 183 in an aqueous soy extract (Cavallini et al., 2011). After 60 days of treatment in rabbits, the administration of the probiotic soy product caused a reduction of the Enterobacteriaceae population (represented mainly by *Escherichia coli*, *Salmonella* spp., *Shigella* spp., *Yersinia enterocolitica*, *Klebsiella* spp., *Proteus* spp., and *Citrobacter* spp.) and a significant increase in *Enterococcus* spp., *Lactobacillus* spp. and *Bifidobacterium* spp., which are known to be beneficial to the host’s health (Mitsuoka, 1990). This effect was due to the presence and activity of *L. helveticus* and *E. faecium*, since the unfermented product did not induce any modification of the gut microbiota. Interestingly, the modification of the microbiota composition was accompanied by an improvement in the lipid profile of the host, measured as a reduction of total cholesterol, non-HDL cholesterol, and auto-antibodies against oxidized LDL; at the same time, HDL cholesterol was increased. In addition, rabbits fed with the probiotic preparation presented a lower extent of atherosclerotic lesion areas in aortic segments. Cavallini et al. (2011) hypothesized that hypocholesterolemic effects induced by bacteria such as Lactobacillaceae and bifidobacteria can occur through various mechanisms, including the deconjugation of bile salts (which cannot be absorbed in the deconjugated form and must be produced again in the liver starting from cholesterol) and the production of propionate and butyrate (which inhibit fatty acid and cholesterol synthesis in the liver; Trautwein et al., 1998). Another study concerning the regulation of lipid metabolism and inflammatory processes was carried out by testing the ability of different LAB to reduce the production of leukotriene B_4_ (LTB_4_; Kimoto-Nira et al., 2009), which is a bioactive lipid generated from arachidonic acid by 5-lipoxygenase and associated with inflammation, allergic reactions, and carcinogenesis when present in excessive amounts. Among a series of LAB tested, *L. helveticus* Bc-10 demonstrated the highest inhibitory capacity on the Ca^2+^ ionophore-induced production of LTB_4_ in the murine macrophage cell line J774.1. Interestingly, heat-inactivated bacterial cells and intracellular cell-free extract maintained the capacity to reduce LTB_4_ expression (Kimoto-Nira et al., 2009).

The administration of *L. helveticus* also affects the metabolic profile of fecal samples. Vitali et al. (2012) analyzed volatile compounds in feces through solid-phase microextraction followed by gas chromatography and mass spectrometry. The results of this study indicated that the administration of *L. helveticus* strain Bar13 to healthy human subjects resulted in a significant increase in butyrate. The production of short-chain fatty acids such as butyrate represented a benefit for gut homeostasis and was likely due to the high secretion of lactate and the consequent conversion of lactate to butyrate by lactate-utilizing bacteria (Duncan et al., 2004). Butyrate is recognized as a specific growth stimulator for colonic epithelial cells and is involved in the reinforcement of barrier integrity, the amelioration of mucosal inflammation, and the oxidative status of the host (Canani et al., 2011). Moreover, butyrate provides protection against colorectal cancer by inducing apoptosis specifically in tumor cells (Hague et al., 1993). In the same study (Vitali et al., 2012), batch culture fermentations of fecal samples by *L. helveticus* Bar13 reduced the fecal concentration of pyridine. Heterocyclic aromatic amines (HCAs) such as pyridine, which are derived from cooking meat and fish, are involved in the etiology of colon cancer in humans (Felton et al., 1997). Several studies reported the ability of LAB to bind HCAs on their bacterial cell wall (Bolognani et al., 1997), preventing the mucosal absorption of these compounds. *L. helveticus* may exert the detoxifying activity observed by Vitali et al. (2012) through an analogous mechanism.

The administration of *L. helveticus* also affects the microbiota composition of the human vaginal mucosa. Treatment with *L. helveticus* KS300 modified the vaginal microbiota by eliciting an increase in Lactobacillaceae and Moraxellaceae and decreasing *Pastereullaceae*, thus counteracting the effects of *C. albicans* on these microbial populations (Atassi et al., 2006).

## *L. helveticus* Modulates Host Immune Responses

Increasing evidence supports the idea that one of the major mechanisms through which beneficial microbes can positively affect the host’s health involves their ability to interact with the host’s immune system by eliciting responses at both the local level and the systemic level (Borchers et al., 2009; Lebeer et al., 2010; Taverniti and Guglielmetti, 2011). Several studies reported the ability of strains belonging to the *L. helveticus* species to exert immunostimulatory effects, both when used alone or in combination with other bacterial strains. For instance, *L. helveticus* strain R0052 was used in association with *Bifidobacterium longum* and *Streptococcus thermophilus* to ferment both a soy beverage and a milk-based blend. When these mixtures were added to the intestinal epithelial cell lines HT29 and T84 before stimulation with tumor necrosis factor (TNF)-α, they significantly decreased the expression of the chemokine interleukin (IL)-8 (Wagar et al., 2009). Furthermore, Ng and Griffiths (2002) observed that cultured macrophages conditioned with *L. helveticus*-fermented milk (LhFM) or its cell-free supernatant were stimulated to secrete IL-6, whereas Laffineur et al. (1996) demonstrated that the cell-free supernatant of *L. helveticus*-fermented β-casein-enriched medium was able to modulate the proliferation of lymphocytes *in vitro*. In another study, mice received milk fermented with *L. helveticus* R389 or the correspondent non-bacterial fraction (NBF; i.e., fermented milk devoid of bacterial cells) before being infected with *Salmonella* Typhimurium. Both treatments caused an increase in immunoglobulin (Ig)-A producing cells in the lamina propria of the small intestine and a higher luminal content of both total S-IgA and specific anti-*Salmonella* IgA (Vinderola et al., 2007b,c). Previously, Leblanc et al. (2004) isolated the peptidic fraction of milk fermented by *L. helveticus* R389 using size-exclusion-HPLC and fed mice the extracts before infecting them with *E. coli* O157:H7. The authors observed an increase in IgA producing B-lymphocytes, total intestinal IgA secretion, and total serum IgA, indicating a stimulation of the systemic immune response. Similarly, Frece et al. (2009) reported that the administration of *L. helveticus* M92, both alone in milk and in a symbiotic preparation, augmented the levels of fecal secretory IgA in mice and total serum IgA. These data suggest that the stimulation of mucosal immunity could play a pivotal role in the protection against pathogens. The administration of milk fermented by *L. helveticus* strains represents a potential alternative treatment for the prevention of enteric infections, which is likely mediated by the production of bioactive compounds. Peptides derived from the high proteolytic activity of *L. helveticus* on food proteins have been demonstrated to stimulate the host’s immune system. A study by Leblanc et al. (2004) showed that the peptides derived from the proteolytic activity of *L. helveticus* R389 on milk induced in a BALB/c murine modela T helper (Th) 2 response, which is an adaptive immune reaction based on humoral responses mounted by a specialized sub-population of T helper lymphocytes. This effect was evidenced by the increased level of serum IL-4, and a concomitant reduction of the pro-inflammatory cell-mediated Th1 response. In another study, three distinct fractions of milk fermented by *L. helveticus* R389, containing different size of peptides (Fraction I, II, and III respectively for large, medium, and small size), were fed to mice that were subsequently treated with methylcholanthrene crystals to induce tumor formation. All fractions tested caused an increase in IgA + B cells in gut-associated lymphoid tissue (GALT), with Fractions II and Fraction III showing a stronger effect, likely due to the presence of a high percentage of l-tryptophan (LeBlanc et al., 2002). Diets supplemented with the peptide fractions also ameliorated tumor progression, as revealed by a reduction in the induced fibrosarcoma volume. The authors suggested that the observed effect could be mediated by the induction of cytokine production from cells involved in inflammatory responses (Perdigón et al., 1999).

Since the GALT is connected to other mucosal-associated lymphoid tissues (MALT), the activation of B and T cells can induce their migration to other sites depending on the type of stimulus, resulting in a systemic modulation of the immune response. Accordingly, Matar et al. (2001) found an induction of IgA-secreting cells both in the small intestine and in the bronchus of mice fed milk fermented by *L. helveticus* R389. In mice, the oral administration of milk fermented by a wild-type strain and a non-proteolytic variant stimulated the immune system and had antitumoral effects, as demonstrated by the regression of a subcutaneously implanted fibrosarcoma. Interestingly, the administration of milk fermented by both strains caused activation of the phagocytic activity of peritoneal macrophages, whereas the increase of IgA-secreting cells in the gut and bronchus was not significant when the non-proteolytic variant was used. In another study in mice, a delay in breast cancer growth was observed after the administration of either *L. helveticus* R389 or kefir fermented by the same strain (de Moreno de LeBlanc et al., 2006). The results demonstrated that both the probiotic strain alone and the fermented product were able to positively affect murine health, even in mucosal sites distant from the direct site of action, resulting in a systemic immune activation.

In a similar study, a 7-days administration of two milk preparations fermented by strains *L. helveticus* 389 and L89 resulted in breast tumor regression in mice. In particular, the administration of milk fermented with strain 389 increased IL-10 and decreased of IL-6 levels, both in the serum and in the mammary glands. Based on these results, the authors suggested that *L. helveticus* 389 may have an effect in the link between immune and endocrine responses (de Moreno de LeBlanc et al., 2005).

In another study, the NBF of milk fermented with *L. helveticus* R389 was fed to BALB/c mice (Vinderola et al., 2007a). In mice that received a diet supplemented with the NBF preparation, a non-specific stimulation of the immune system occurred (MacPherson et al., 2000; Fagarasan et al., 2001), as demonstrated by an increase of IgA + cells in the small intestine and by the higher production of IL-2, IL-6, and total secretory IgA in the lumen during the feeding period. The beneficial effects could be linked to IL-6 and IgA, which are known to be responsible for improved mucosal homeostasis and immunological defense (Brandtzaeg et al., 1987).

It is important stress that in the above mentioned study by Vinderola et al. (2007a), NBFs were prepared by simple centrifugation; therefore, it is not possible to claim that the observed activity was due to milk-derived bioactive peptides. Recently, Stuknyte et al. (2011) observed a clear reduction of the immunomodulatory properties of casein hydrolyzates (CHs) when the bacterial cells of *L. helveticus* MIMLh5 (used for the digestion of casein) were removed by ultrafiltration through a 3 kDa-cut off membrane but not by centrifugation. Compared to whole CHs, the cell-free ultra-filtrate did not reduce the activation of NF-κB in the reporter system based on recombinant intestinal epithelial Caco-2 cells, indicating that the immunomodulatory activity of strain MIMLh5 was very likely dependent on the presence of bacterial cells rather than on the production of casein-derived bioactive peptides.

The same strain of *L. helveticus*, MIMLh5, has recently been demonstrated to exert immunomodulatory effects on NF-κB activation at the oro-pharyngeal level. These experiments used an *in vitro* model based on the hypopharyngeal FaDu cell line, both at baseline and in presence of IL-1β, a cytokine used to mimic a pro-inflammatory stimulus (Guglielmetti et al., 2010b). Since NF-κB is a transcriptional factor involved in the activation of several pro-inflammatory cytokines (Baldwin, 1996) and a therapeutic target in several human (auto) inflammatory diseases (Yamamoto and Gaynor, 2001), the cytokine profile triggered by *L. helveticus* MIMLh5 on FaDu cells was analyzed. Strain MIMLh5 induced a different profile depending on the absence or presence of IL-1β. At baseline, MIMLh5 significantly reduced the levels of pro-inflammatory cytokines TNF-α, IL-8, and IL-6. In the presence of IL-1β, MIMLh5 increased IFN-γ, GM-CSF, and IL-6. A possible explanation for this different behavior may lie in the activation of a heat-shock response, mediated by the bacterial induction of the heat-shock protein gene *hsp70* that is known to exert protective effects on cytokine transcripts (Laroia et al., 1999). This explanation is also consistent with the reduction of NF-κB activation (Bao and Liu, 2009). The different effects of *L. helveticus* on IL-6, a pleiotropic cytokine associated with both pro-inflammatory (Papanicolaou et al., 1998) and anti-inflammatory events (Xing et al., 1998), is particularly interesting. *L. helveticus* MIMLh5 also increased the levels of GM-CSF, a factor required for myeloid dendritic cell (DC) survival, leading to a higher GM-CSF/G-CSF ratio. This ratio is associated with a switch of the immune response to a Th1 profile, which is also consistent with the observed induction of TNF-α and IL-2 in bone marrow-derived DCs stimulated with MIMLh5 (Guglielmetti et al., 2010b). These results suggest that MIMLh5 can potentially exert immunomodulatory effects on pharyngeal cells and elicit systemic immune responses. Activated DCs, together with the production of IL-2 (Granucci et al., 2003), are necessary to prime natural killer (NK) cells to produce IFN-γ and to induce a Th1 response. In a subsequent study, strain MIMLh5 was tested together with the oral isolate *S. salivarius* ST3 (Guglielmetti et al., 2010a) on the human macrophage U937 cell line (Taverniti et al., 2012). The combination of MIMLh5 and ST3 elicited a balanced ratio of TNF-α and IL-10 that might be produced by a mild activation of the immune system and may be useful for combating infections, potentially without causing a detrimental outcome. Moreover, the combined administration of the two strains affected the activation of COX2, an enzyme involved in the synthesis of prostaglandins and in inflammatory processes (Funk, 2001). COX2, has recently been demonstrated to have a positive impact on the host’s health because of its role in inducing oral tolerance, resolving inflammation, and restoring mucosal integrity (Williams et al., 1999; Morteau et al., 2000).

Another example of the immunomodulatory properties of *L. helveticus* outside the gut was reported for strain HY7801, which was demonstrated to modulate the immune response in the vagina. The oral administration of *L. helveticus* HY7801 in *C. albicans*-infected mice induced a reduction of NF-κB activation in vaginal tissue, decreased the expression of IL-1β, TNF-α, IL-6, COX2, and iNOS, and increased the expression of IL-10. Moreover, the same anti-inflammatory activity was confirmed in peritoneal macrophages, since *L. helveticus* efficiently counteracted the LPS-induced inflammatory response in macrophages isolated from mouse peritoneal cavities (Joo et al., 2012).

In summary, several *in vitro* and *in vivo* studies confirmed that specific strains of *L. helveticus* can exert significant beneficial immunomodulatory properties. *L. helveticus* can act on the intestine, the vaginal mucosa, or the pharyngeal epithelium, through application of bacterial cells, bacterial cell components, or bacterially produced bioactive molecules. In conclusion, *L. helveticus* has the ability to support cross-talk with host’s immune system, potentially causing beneficial outcomes at the local and systemic levels.

## Other Proposed Health-Promoting Properties of *L. helveticus*

Several studies suggested that strains of *L. helveticus* can influence several different aspect of the host’s physiology. In a study performed by Matar et al. (1997), milk fermented by *L. helveticus* L89 and filtered through a 0.22 μm microporous membrane showed an inhibitory effect on mutagenesis induced by 4-nitroquinoline-*N*′-oxide in the Ames genotoxicity test (Maron and Ames, 1983). On the contrary, milk fermented by the non-proteolytic variant Prt^−^ of the same *L. helveticus* strain did not have significant activity, suggesting that peptides released during fermentation are responsible for the antimutagenic effect.

Vinderola et al. (2007a) observed that the administration of the NBF of milk fermented by *L. helveticus* R389 caused a transient activation of calcineurin (a phosphatase involved in the regulation of both Ca^2+^ pumps and exchangers for the maintenance of Ca^2+^ homeostasis; Bandyopadhyay et al., 2002) and enhanced the transcription of the T cell stimulating factor IL-2. The administration of NBF to mice also caused increased expression of the transient receptor potential channel (TRP) V6, a specific channel for Ca^2+^, which induced an increase of Ca^2+^ uptake. Furthermore, NBF administration increased the number of local mast cells and goblet cells, which take part in providing epithelium protection through the production of mucus. In summary, administration of NBF resulted in an improvement of the host’s nutritional status and defense due to improved intestinal barrier functions and surveillance. The effect of *L. helveticus* supplementation on nutritional status has been also documented in other studies. For instance, the administration of a multi-strain probiotic preparation containing *L. helveticus* strain LAT 179 to broiler chickens caused an increase in body weight and serum albumin levels, an increase in serum levels of minerals (i.e., calcium and potassium), a decrease in triglyceride levels and an improved antioxidant status (Capcarova et al., 2011). In another study, the administration of an aproteic diet with *L. helveticus* Bionan (Nestlé, Brazil) in combination with *S. thermophilus* protected the colonic mucosa of mice by counteracting the effect of malnutrition and restoring the number of goblet cells (Dock-Nascimento et al., 2007). Analogously, the same probiotic preparation administered to mice in pre- and post-operative periods stimulated the immune system, increased the production of serum IgA and increased colonic weight (Aguilar-Nascimento et al., 2006).

Fermented milk whey containing *L. helveticus* also proved beneficial in ameliorating induced dermatitis in a murine model. The oral administration of milk fermented with *L. helveticus* strain CM4 decreased transepidermal water loss and the size of dermatitis area in SDS-exposed Hos:HR-1 hairless mice, improving skin moisture content. On the contrary, no significant effects were exerted by artificially acidified milk whey (Baba et al., 2010). *In vitro* experiments on human epidermal keratinocytes supplemented with the same fermented milk whey preparation identified an increase in profilaggrin mRNA, a precursor of filaggrin that is involved in epidermal hydration and flexibility and is a marker of epidermal terminal differentiation (Baba et al., 2006).

Oral administration of *L. helveticus* R0052 and *B. longum* R0175 (formulation PF; Probio’Stick, Institute Rosell-Lallemand, Montreal, QC, Canada) to mice after induced myocardial infarction (MI) was effective in attenuating the resulting symptoms of depression (Arseneault-Bréard et al., 2012). Feeding with PF improved intestinal barrier integrity and depression symptoms, measured by various behavioral and social interaction tests performed on mice. Moreover, PF administration induced a reduction in circulating IL-1β levels (Arseneault-Bréard et al., 2012). These data suggest that *L. helveticus* species can potentially influence the gut-brain axis and thus affect the central nervous system (Neufeld and Foster, 2009), as also proposed by studies of the connection between stress and gastrointestinal disorders (Forsythe et al., 2010). The same probiotic formulation containing *L. helveticus* R0052 and *B. longum* R0175 (PF) attenuated apoptosis in the limbic region (involved in the pathophysiology of depression) that can occur after MI, measured by a reduction in caspase-3 activation (Girard et al., 2009). This effect was hypothesized to be due to a reduction of pro-inflammatory cytokines, which can be involved in apoptosis (Wann et al., 2006). Administration of PF was also effective in attenuating anxiety-like behavior in rats and anxiety symptoms in normal volunteers who took PF daily for 30 days. Volunteers scored lower on the Hopkins Symptoms Checklist (HSCL)-90, a 90-item self-reported questionnaire that evaluates several behavioral and emotional parameters, such as somatization, depression, and anger-hostility. It has been hypothesized that probiotics may exert their anxiolytic effects by competing with pathogens, since gastrointestinal infections seem to affect the brain area that connects viscerosensory information and mood via the vagus nerve, leading to changes in neurotransmitter levels and functions.

Beneficial effects of *L. helveticus*-fermented products were also reported on sleep quality. A preparation obtained through the fermentation of milk with *L. helveticus* CM4 administered for 3 weeks to healthy elderly people resulted in a decrease in the number of wake episodes and a global improvement of sleep quality, as revealed by a questionnaire and confirmed by actigraphy analysis. The effect was suggested to be due to the presence of a tripeptide produced by *L. helveticus* during milk fermentation that inhibits angiotensin-I-converting enzyme (ACE) activity (consistent with the fact that certain antidepressant drugs act as inhibitors of ACE; Zubenko and Nixon, 1984) or, more likely, to the production of bioactive peptides that act on GABAergic or serotonergic neurons (Yamamura et al., 2009).

## ACE Inhibitory Activity

Angiotensin-I-converting enzyme (ACE; peptidyl-dipeptide hydrolase, EC 3.4.15.1) is a dipeptidyl carboxypeptidase located in different tissues of the human body. ACE is responsible for increasing blood pressure by converting angiotensin-I to the potent vasoconstrictor angiotensin-II and by degrading the vasodilator bradykinin (Campbell, 1987). Several studies demonstrated that fermentation and/or digestion processes liberate small peptides from several food proteins that inhibit the ACE activity (Abubakar et al., 1998; Dziuba et al., 1999; Fitzgerald and Meisel, 2000; Gobbetti et al., 2000). Interest in ACE inhibitory peptides was amplified by the experimental observation that they may exert an antihypertensive effect after oral administration (Vermeirssen et al., 2004). A large number of studies proved that among LAB, *L. helveticus* has the highest capacity to form ACE inhibitory peptides from food proteins as a consequence of the marked activity of its cell envelope-associated proteinases (CEPs; Yamamoto et al., 1993). The first observations of the ability of *L. helveticus* to generate ACE inhibitory peptides date back to the nineties, when Yamamoto et al. (1994a) showed that the peptides liberated from casein by the CEP activity of *L. helveticus* CP790 were able to suppress ACEs. The same authors found that the ACE inhibitory activity was higher in the whey fractions of milk fermented with *L. helveticus* than in milk fermented with other LAB, including *L. delbrueckii*, *L. casei*, *Streptococcus thermophilus*, *Lactococcus lactis* and, notably, *L. acidophilus* (Yamamoto et al., 1994b). Accordingly, they observed that fermented milk, upon oral administration, significantly lowered blood pressure in spontaneously hypertensive rats only if the milk was prepared with *L. helveticus* strains and not with other LAB (Yamamoto et al., 1994b).

The ACE inhibitory activity of milk fermented with *L. helveticus* has been linked to several different peptides, mainly originating from the digestion of the αs1, β, and κ fractions of casein (Takano, 1998). Specifically, the following amino acidic sequences have been associated with ACE inhibitory peptides: Ala-Tyr-Phe-Tyr-Pro-Glu, Ser-Lys-Val-Leu-Pro-Val-Pro-Gln (Yamamoto et al., 1994a), Val-Pro-Pro and Ile-Pro-Pro (Nakamura et al., 1995), Lys-Val-Leu-Pro-Val-Pro-Gln (Maeno et al., 1996), and Tyr-Pro (Yamamoto et al., 1999).

More recently, it was demonstrated that the *L. helveticus* strain LMG11474 can produce considerable amounts of ACE inhibitory peptides by the fermentation of pea proteins (Vermeirssen et al., 2003). However, the ability of *L. helveticus* to generate ACE inhibitory peptides from food proteins other than casein has only been marginally investigated.

Many *in vivo* trials assessed the suitability of LhFM as an additional or alternative treatment for hypertension. The ability of LhFM to reduce blood pressure has been demonstrated in several studies employing a spontaneously hypertensive rat model (Yamamoto et al., 1994b; Takano, 1998; Sipola et al., 2001; Jakala et al., 2009). Moreover, promising results supporting the ability of LhFM to reduce blood pressure have also been obtained in human clinical studies. A meta-analysis of 12 randomized controlled trials published in 2008 provided evidence that fermented milk has hypotensive effects in both prehypertensive and hypertensive subjects. Interestingly, nine out of the 12 randomized controlled trials included in the meta-analysis were based on milk fermented with *L. helveticus* (Xu et al., 2008).

More recent double-blind, randomized, placebo-controlled, parallel group studies showed that the long-term (3 months) intake of milk fermented by *L. helveticus* (strain LBK-16 H) containing Ile-Pro-Pro and Val-Pro-Pro tripeptides reduced systolic and diastolic blood pressure in 24-h readings. The differences between the treatment group and the placebo group were not statistically significant, but the authors concluded that the observed effect may have clinical significance (Jauhiainen et al., 2010, 2012). In addition, Usinger et al. (2010) recently reported that LhFM (strain Cardi04) did not inhibit ACE in humans. Furthermore, they reported that 24-h ambulatory blood pressure measurements failed to reveal significant antihypertensive effects during an 8-week intervention study based on the consumption of 150 or 300 ml of LhFM, even though the group receiving 300 ml of LhFM had reduced blood pressure across the 8-week period in several readings (Usinger et al., 2010). It has been proposed that divergent or statistically insignificant results in human studies can be explained by the marked subject-to-subject variability in the effects of lactotripeptides (Pripp, 2008; Xu et al., 2008; Cicero et al., 2011), which may mask the effects of clinically significant mean blood pressure reductions (Jauhiainen et al., 2012).

In conclusion, results from human interventional trials indicate that LhFM produces at least mild antihypertensive effects.

## Indirect Health-Promoting Effects of *L. helveticus*

In addition to the production of ACE inhibitory peptides from food proteins, *L. helveticus* can indirectly benefit the host’s health by reducing allergens or toxic compounds and increasing the bioavailability of specific nutrients in foodstuffs.

Stidl et al. tested the capacity of eight LAB species (12 strains per species) to inactivate five heterocyclic aromatic amines (HACs), including the molecules 2-amino-1-methyl-6-phenyl-imidazo[4,5-*b*]pyridine and 2-amino-9H-pyrido[2,3-*b*]indole, which are the most abundant mutagens in fried red meat. The inactivation of the amines with LAB resulted in a decrease of their mutagenic potential and correlated with the HCA-binding capacities of the single strains. Notably, this study showed that the overall inactivation of the different HCAs by *L. helveticus* (calculated to be about 83%) was the highest among the tested bacteria, which also included *L. delbrueckii* susp. *bulgaricus* (59%) and *L. acidophilus* (20%; Stidl et al., 2008).

In a subsequent study, *L. helveticus* fermentation was shown to significantly decrease the antigenicity of whey proteins in skim milk. Bu et al. (2010), using indirect competitive ELISA, observed that strain *L. helveticus* B1, alone or in combination with *S. thermophilus*, was the most effective in reducing the antigenicity of α-lactalbumin and β-lactoglobulin, the major allergens in cow’s milk.

More recently, *L. helveticus* was successfully employed in a biotransformation aimed to remove allergenic molecules from propolis (bee glue). *Propolis* is a resinous, sticky, dark-colored material produced by honeybees (*Apis mellifera*), which is extensively used as a natural ingredient in food formulations and supplements. A study by Gardana et al. (2012) demonstrated that the cinnamoyl esterase activity of *L. helveticus* strains MIMLh5 and SLh02 can significantly degrade the major allergens in propolis, including esters of caffeic acid such as methyl-butenyl-caffeates.

In a previous study, the strong cinnamoyl esterase activity of *L. helveticus* strain MIMLh5 was employed to ferment Renetta cultivar apple pulp with the aim of converting the chlorogenic acid into free caffeic acid. Caffeic acid and chlorogenic acid are potent antioxidant molecules that have been associated with several health-promoting effects *in vivo* (Suzuki et al., 2002; Takeda et al., 2002; Touaibia et al., 2011; Weng and Yen, 2012). Nonetheless, the biological properties of these hydroxycinnamic acids depend on their absorption by the gastrointestinal tract. Whereas the majority of chlorogenic acid reaches the large intestine and is quickly degraded by the microbiota, free caffeic acid is well absorbed in the stomach and small intestine, resulting in a higher plasma concentration of this molecule and its metabolites than chlorogenic acid. Therefore, the fermentation of Renetta apple pulp by *L. helveticus* strain MIMLh5 could significantly increase the bioavailability of hydroxycinnamic acids by preserving the overall antioxidant power of apple pulp (Guglielmetti et al., 2008).

The capacity of *L. helveticus* to increase the bioavailability of specific nutrients was also demonstrated with soy isoflavones (SIs). SIs are polyphenolic compounds with estrogen-like effects, which have been associated with health benefits as a consequence of their antioxidant (Ruiz-Larrea et al., 1997) and anti-inflammatory activities (Huang et al., 2005). The main isoflavones of soy (daidzin, genistin, glycitin) are naturally present as β-glucosides, which are not readily bioavailable in humans since they are very poorly absorbed through the intestinal tract (Donkor and Shah, 2008). The γ-glucosidase activity of *Lactobacillus helveticus* R0052 during soy fermentation can convert isoflavone glucosides into aglycone forms, improving the bioavailability of these molecules (Champagne et al., 2010).

In conclusion, the few examples that we mentioned demonstrate that *L. helveticus* strains possess enzymes, such as CEPs, cinnamoyl esterase, or β-glucosidase, which can contribute to the improvement of food quality and safety.

## Conclusion

*L. helveticus* is a “Generally Recognized as Safe” (GRAS) microorganism, which was given the “Qualified Presumption of Safety” (QPS) status by the European Food Safety Authority (EFSA) (2007). The QPS is the EFSA’s safety evaluation label based on “the body of knowledge” or “familiarity” of a microorganism. Therefore, *L. helveticus* deserves QPS status, since it has a long history of apparent safe use in the food chain, is sensitive to most antibiotics and lacks acquired antibiotic resistance (Rossetti et al., 2009; Guglielmetti et al., 2010b).

In addition to the mandatory requirements for safety (Food and Agriculture Organization/World Health Organization, 2002), a microbial strain has to demonstrably benefit the host’s health in order to be considered a probiotic, according to the currently accepted definition. In this manuscript, we reviewed the literature indicating that different *L. helveticus* strains can positively influence the host’s physiology and prevent or treat pathologic conditions. Several mechanisms of action have been proposed by *in vitro* studies, such as the direct inhibition of pathogens, the stimulation of DCs and macrophages, or the modulation of host gene expression. Furthermore, *in vivo* clinical and interventional trials have substantiated the probiotic efficacy of *L. helveticus*.

In conclusion, this review demonstrated that strains belonging to the *L. helveticus* species can display probiotic properties that are not dissimilar from those displayed by microorganisms conventionally considered to be probiotics, such as *L. acidophilus*, *L. rhamnosus*, and *Bifidobacterium animalis* subsp. *lactis*. Additionally, properties such as the production of ACE inhibitory peptides or cinnamoyl esterase activity are unique to *L. helveticus* strains and provide support for use of this bacterium over other probiotic species. *L. helveticus* can adapt to industrial fermentation conditions more easily than most intestinal probiotic lactobacilli and bifidobacteria, due to its ability to survive various environmental stresses such as high temperatures or low pH, osmotic pressure, and oxygen, which facilitates its incorporation into new probiotic formulas.

Considering the existing scientific knowledge on the health-promoting properties of *L. helveticus* (Table 1), this bacterial species can be properly considered to be probiotic.

**Table 1:** **Reported health-promoting activities displayed by bacterial strains belonging to *Lactobacillus helveticus* species**.

*L. helveticus* Strain	Health-promoting agent	Activity	Type of study	Reference
R389	Bacterial cells	Protective action against *Salmonella enteritidis* serovar Typhimurium infection	*In vivo*, mouse	Vinderola et al. (2007b)
R0052	Viable and heat-inactivated bacterial cells	Reduction of *Campylobacter jejuni* invasion in human colon cancer epithelial T84 cells	*In vitro*	Ternhag et al. (2008)
	Bacterial cells in association with *Lactobacillus rhamnosus*	Amelioration of mucosal barrier functions and colonic epithelial cells hyperplasia in mice infected with *Citrobacter rodentium*	*In vivo*	Gareau et al. (2010)
	Bacterial cells	Blocking of innate immunity pathogen-subversion by interfering with *E. coli* O157:H7-induced inhibition of STAT-1 tyrosine phosphorylation in 407, Caco-2 and HEp cells	*In vitro*	Jandu et al. (2009)
	Isolated S-layer protein	Inhibition of *E. coli* O157:H7 adhesion on intestinal epithelial HEp-82 cells and pathogen-induced drop in trans-epithelial resistance on T84 cell line	*In vitro*	Johnson-Henry et al. (2007)
M92	Isolated S-layer protein	Mediation of bacterium auto-aggregation and co-aggregation with *Salmonella* Typhimurium FP1	*In vivo*, mouse	Beganovic et al. (2011)
MIMLh5	Bacterial cells	Adhesion to human hypopharyngeal epithelial FaDu cells and HaCat keratinocytes; competitive exclusion against *Streptococcus pyogenes*	*In vitro*	Guglielmetti et al. (2010b)
KS300	Bacterial cells	Inhibition of growth and viability of vaginosis-associated bacteria and pathogen adhesion on human cervical Hela and Caco-2/TC7 enetrocyte-like cell lines	*In vitro*, human	Atassi et al. (2006)
HY7801	Bacterial cells	Inhibition of *Candida albicans*-induced vaginitis and reduction of myeloperoxidase activity in vaginal tissue	*In vivo*, mouse	Joo et al. (2012)
M92	Bacterial cells alone and in association with prebiotic fibers	Increase of total lactic acid bacteria (LAB) fecal counts; reduction of total enterobacteria and sulfur-reducing clostridia levels in fecal samples	*In vivo*, mouse	Frece et al. (2009)
	Bacterial cells	Decrease of total enterobacterial and *Salmonella* spp. fecal counts	*In vivo*, mouse	Beganovic et al. (2011)
416	Association with *Enterococcus faecium* CRL 183 in aqueous soy extract	Reduction of the Enterobacteriaceae population, increase in *Enterococcus* spp., *Lactobacillus* spp. and *Bifidobacterium* spp. fecal counts. Amelioration of lipid profile. Reduction of atherosclerotic lesion area in aortic segments	*In vivo*, rabbit	Cavallini et al. (2011)
Bc-10	Viable and heat-inactivated bacterial cells; intracellular cell-free extract	Inhibition of leukotriene B_4_ production on J774 cell line	*In vitro*, mouse	Kimoto-Nira et al. (2009)
Bar13	Bacterial cells	Increase in butyrate and decrease in pyridine levels in fecal samples	*In vitro*, humans	Vitali et al. (2012)
KS300	Bacterial cells	Increase in Lactobacillaceae and Moraxellaceae vaginal levels and decrease in Pastereullaceae population	*In vitro*, human	Atassi et al. (2006)
R0052	Bacterial cells in association with *Bifidobacterium longum* and *Streptococcus thermophylus* in soy and milk fermented preparations	Decrease in TNF-α-induced expression of IL-8 in human epithelial HT29 and T84 cells	*In vitro*	Wagar et al. (2009)
LH-2	Whole fermented milk and cell-free supernatant	Stimulation of IL-6 secretion in macrophages	*In vitro*	Ng and Griffiths (2002)
5089	Ultra-filtered fermented β-casein-permeate	Proliferation of human peripheral-blood lymphocytes	*In vitro*	Laffineur et al. (1996)
R389	Whole fermented milk and non-bacterial fraction	Increase in immunoglobulin (Ig)-A producing cells in the lamina propria and in luminal content of total S-IgA and specific anti-*Salmonella* IgA	*In vivo*, mouse	Vinderola et al. (2007c)
	Peptidic fraction of fermented milk	Increase of IgA producing B-lymphocytes, total intestinal IgA secretion and total serum IgA in mice infected with *E. coli* O157:H7	*In vivo*	Leblanc et al. (2004)
M92	Milk supplemented with viable bacterial cells alone and in association with prebiotics	Augmentation of fecal secretory IgA levels and total serum IgA in mice	*In vivo*	Frece et al. (2009)
R389	Peptidic fraction of fermented milk	Induction of a Th2 responses (high IL-4 serum levels)	*In vivo*, mice	Leblanc et al. (2004)
	Different peptide size fractions from fermented milk	Increase of IgA + B cells at mucosal gut level; amelioration of induced-tumor in mice	*In vivo*	LeBlanc et al. (2002)
	Fermented milk	Induction of IgA-secreting cells in small intestine and in bronchus in mice	*In vivo*	Matar et al. (2001)
	Milk fermented with wild-type and a non-proteolytic variant	Regression of a subcutaneous implanted-fibrosarcoma in mice; activation of the phagocytic activity in mouse peritoneal macrophages	*Ex vivo, in vivo*	Matar et al. (2001)
	Whole fermented kefir and cell-free kefir	Breast cancer growth delay	*In vivo*, mouse	de Moreno de LeBlanc et al. (2006)
R389; L89	Fermented milk	Breast cancer regression	*In vivo*, mouse	de Moreno de LeBlanc et al. (2005)
R389	Fermented milk	Increase of IL-10 and decrease of IL-6 levels in the serum and in mammary glands	*In vivo*, mouse	de Moreno de LeBlanc et al. (2005)
R389	Non-bacterial fraction of fermented milk	Increase of IgA + cells in small intestine, production of IL-2, IL-6, and total secretory IgA in the lumen	*In vivo*, mouse	Vinderola et al. (2007a)
MIMLh5	Casein hydrolyzates	Reduction of NF-κB activation in human epithelial Caco-2 cells	*In vitro*	Stuknyte et al. (2011)
	Bacterial cells	Reduction of NF-κB activation and induction of distinct cytokine profile on human FaDu cells in different physiological conditions; stimulation of TNF-α and IL-2 production in mouse bone marrow-derived dendritic cells	*In vitro, ex vivo*	Guglielmetti et al. (2010b)
	Bacterial cells in association with *Streptococcus salivarius* ST3	Stimulation of innate immune responses in human macrophages U937 cells	*In vitro*	Taverniti et al. (2012)
HY7801	Bacterial cells	Reduction of NF-κB and pro-inflammatory cytokines expression in *Candida albicans*-infected mice; anti-inflammatory action on LPS-stimulated peritoneal macrophages	*Ex vivo, in vivo*	Joo et al. (2012)
L89	Ultra-filtered fermented milk	Inhibition of induced-mutagenesis (Ames test)	*In vitro*	Matar et al. (1997)
R389	Non-bacterial fraction of fermented milk	Improvement of Ca^2+^ uptake through activation of calcineurin and TRPV6 channel; increase of mast cells and goblet cells number	*In vivo*, mice	Vinderola et al. (2007a)
LAT179	Bacterial cells in a multi-strain probiotic preparation	Increase of body weight, albumin serum level and amelioration of mineral parameters, antioxidant status and lipid profile in broiler chickens	*In vivo*	Capcarova et al. (2011)
Bionan	Bacterial cells in association with *S. thermophylus*	Protection of colonic mucosa in mice fed with aproteic diet	*In vivo*	Dock-Nascimento et al. (2007)
	Bacterial cells in association with *S. thermophylus*	Increase of colonic weight and serum IgA levels in pre and post-operative period	*In vivo*, mice	Aguilar-Nascimento et al. (2006)
CM4	Fermented whey milk	Amelioration of induced dermatitis in mice	*In vivo*	Baba et al. (2010)
	Fermented whey milk	Improvement of epidermal differentiation in human epidermal keratinocytes	*In vitro*	Baba et al. (2006)
R0052	Bacterial cells in a probiotic preparation with *B. longum*	Attenuation of post-myocardial infarction depression symptoms in mice	*In vivo*	Arseneault-Bréard et al. (2012)
	Bacterial cells in a probiotic preparation with *B. longum*	Reduction of apoptosis in limbic region in rats	*In vivo*	Girard et al. (2009)
	Bacterial cells in a probiotic preparation with *B. longum*	Anxiolytic effect in rats; attenuation of anxiety symptoms in human volunteers	*In vivo*	Messaoudi et al. (2011)
CM4	Fermented milk	Amelioration of sleep quality in elderly people	*In vivo*, humans	Yamamura et al. (2009)
CP790	Casein hydrolyzates	Angiotensin-I-converting enzyme inhibitory (ACEI) activity, decrease of blood pressure in spontaneously hypertensive rats	*In vivo*	Yamamoto et al. (1994a)
LMG11474	Fermented-pea proteins	Release of ACE inhibitory peptides (ACE inhibition assay)	*In vitro*	Vermeirssen et al. (2003)
*LBK-16 H*	Fermented milk	Reduction of systolic and diastolic blood pressure	*In vivo*, humans	Jauhiainen et al. (2010, 2012)
MVLH1; MVLH2	Bacterial cells	Inactivation of heterocyclic aromatic amines	*In vitro*	Stidl et al. (2008)
B1	Bacterial cells alone and in association with *S. thermophylus* in milk	Decrease of antigenicity of caw milk α-lactalbumin and β-lactoglobulin	*In vitro*	Bu et al. (2010)
MIMLh5; SLh02	Cinnamoyl esterase activity	Reduction of allergenic caffeic acid esters content in propolis	*In vitro*	Gardana et al. (2012)
MIMLh5	Cinnamoyl esterase activity	Improvement of antioxidant potential of apple pulp through increase of hydroxycinnamic acids bioavailability	*In vitro*	Guglielmetti et al. (2008)
R0052	γ-glucosidase activity	Increase of isoflavones bioavailability and antioxidant activity	*In vitro*	Champagne et al. (2010)

## Conflict of Interest Statement

The authors declare that the research was conducted in the absence of any commercial or financial relationships that could be construed as a potential conflict of interest.
